# Response to the letter to the Editor by Professors Francesco Giammarile and Stefano Fanti regarding our article “Survey by the ANSM of the imaging protocol, detection rate, and safety of ^68^Ga-PSMA-11 PET/CT”

**DOI:** 10.1007/s00259-021-05393-1

**Published:** 2021-05-31

**Authors:** Yanna-Marina Chevalme, Lotfi Boudali, Marc Janier, Jean-Noël Talbot

**Affiliations:** 1 Direction des médicaments en oncologie, hématologie, transplantation, néphrologie, thérapie cellulaire, produits sanguins, et radiopharmaceutiques, Agence Nationale de Sécurité du Médicament et des produits de santé (ANSM), ANSM, 143 Bd Anatole France, 93200 St Denis, France; 2 Comité Scientifique Permanent de l’ANSM: Médicaments de diagnostic et de médecine nucléaire (MDMN), St Denis, France

Dear Sir,

The letter to the Editor of EJNMMI issued by Professors Francesco Giammarile and Stefano Fanti [1] regarding our “Survey by the ANSM of the imaging protocol, detection rate, and safety of ^68^Ga-PSMA-11 PET/CT” [2] offers us the opportunity to lift some misunderstandings. The major part of their letter addresses the medical and regulatory context of our survey, rather than its results. In our opinion, it reflects some confusion between the roles of marketing authorisation (MA), of compassionate use under the strict surveillance of ANSM in France or equivalent medicine agencies in EU for non-registered medications including diagnostic agents, of Guidelines issued by professional societies, and of clinical trials sponsored by industrial firms or by academic or clinical research bodies. We will briefly clarify these points and identify which of them apply to our survey in the case of the diagnostic radiopharmaceutical agent, ^68^Ga-PSMA-11, a ligand of the prostate-specific membrane antigen (PSMA) for PET imaging.

ANSM, the French National Agency for Medicines and Health Products, is a public establishment under the authority of the French Ministry of Health. On behalf of the French government, it is responsible for the safety of health products and promotes access to therapeutic and diagnostic innovation.

As early as 2010, ANSM granted a MA to ^18^F-fluorocholine (FCH) on basis of a prospective pivotal study comparing its diagnostic performance to that of ^18^F-sodium fluoride (registered in France in 2008) for the detection of bone metastases in prostate cancer, using PET. On the following years, this indication was extended, based on the results of further trials, to the detection of distant spread of prostate cancer, in particular in case of biochemical recurrence (BCR). The MA of FCH does not mention any “cut-off” value for serum level of prostate-specific antigen (PSA) in case of BCR.

As a derogation to the MA procedure, Article L.5121-12 of the French Code of Public Health sets exceptional regulations governing the use of medicinal products without a MA in France which are not available in clinical trials and which are intended to treat or to diagnose serious or rare diseases, when no appropriate treatment or diagnostics exist and the initiation of treatment or diagnostic cannot be deferred. After a careful examination by ANSM assessing that the efficacy/safety ratio based on available data may be presumed to be favourable in the claimed indication, this official procedure may result in granting a Temporary Authorisation for Use (ATU). A nominative ATU (nATU) can be requested by a physician and a pharmacist (respectively a specialist of nuclear medicine and a radiopharmacist in the present case) under their responsibility, for a single identified patient who cannot participate in a clinical trial.

Concerning a ^68^Ga-PSMA-11 ligand, the first request for a nATU in view of PSMA-11 PET/CT at Hôpital Tenon in Paris was issued at the beginning of 2016, for a patient with BCR of a prostate cancer. ANSM checked that the regulatory conditions were met:

- Data from literature comparing the results of PET/CT in BCR patients using a PSMA ligand vs. FCH evidenced that the efficacy/safety ratio may be presumed to be favourable for such ligand [3, 4].
- FCH PET/CT has been performed in this patient, which yielded a non-conclusive result that did not contribute to patient’s management and left no other accurate alternative for PET with a registered tracer.
- The radiopharmacy of the PET centre was able to label PSMA-11 with the eluate of a MA registered ^68^Ge/^68^Ga generator, according to the good manufacturing practices.

In conformity with the principle of equality between the patients, nATUs were then granted for other patients from other PET centres who matched all those conditions.

In the nATU agreement, it was specified that the applicant must return information to ANSM about the safety and efficacy of this diagnostic agent observed in each patient. In our survey, information obtained during the first 3 years of issuing those nATUs was gathered, checked and analysed by ANSM.

This survey benefits from a prospective recruitment monitored by ANSM, an independent official body (all observations must be forwarded to ANSM, no way to ignore a “non-conform” result), in a homogeneous setting: BCR of prostate cancer with non-conclusive PET using a radiopharmaceutical registered in this indication. But, according to the regulatory framework recalled above, this is not a comparative study, neither designed nor “sponsored” by ANSM. Actually two direct comparative studies [3, 4], aiming at establishing the superiority of a PSMA ligand over FCH in the detection of BCR were already published in 2016 and formed the basis to accept nATUs for PSMA-11. Pooling those two series, PET/CT showed foci in 57/75 = 76% of patients with the PSMA ligand vs. 38/75 = 51% with FCH. Another article [5] reported, in 32 BCR patients selected on basis of a negative PET/CT with FCH, evocative foci visualised with the PSMA ligand in 14/32 = 44% cases. Therefore, in addition to matching the criteria for nATU, there was a justification (in the radiation protection meaning of this term) for doubling the radiation exposure from PET/CT in those patients, which was not the case for FCH-positive patients, at least according to the data available at that time.

The main objective of our survey, gathering results obtained in the context of clinical routine, was to confirm on a large series that this justification was valid not only in patients with serum PSA levels <1 ng/mL, in whom the detection rate of FCH was known to be around 30% [6], but also in patients with serum PSA levels >2 ng/mL. In those latter cases, FCH has a good detection rate (around 80% [6]) and it would have been possible that, in a large number of them, a non-conclusive FCH PET/CT would be followed by a similar result with ^68^Ga-PSMA-11.

As remarked by Professors Giammarile and Fanti, serum PSA levels were < 1 ng/mL in 356 cases. They recall that, *“in 2015, the guideline for prostate cancer was endorsed by the European Association of Urology (EAU) which, for BCR imaging with choline PET, suggested a cut off value between 1 and 2 ng/mL”*. But, as they recognise, it is clear that a professional guideline, even international, *“has no binding value over procedures of National Agencies”*. Actually, the positivity rate of FCH in BCR below a serum PSA level of 1 ng/mL is limited but not nil, 66/211 = 31%, and 18/211 = 9% of doubtful results [6]. Furthermore, some cases of recurrent prostate cancers which do not overexpress PSMA but take-up FCH have been described [7] and ANSM must not take the risk of a loss of chance for those patients by accepting to skip performing PET/CT with the registered agent FCH.

The authors of the letter also noted that 17 of the patients had a PSA < 0.2 ng/mL, “*thus being even questionable regarding the BCR status.*” In our survey, ^68^Ga-PSMA-11 PET/CT was positive in 7/17 = 41% of those cases. They stated that “*the BCR status could also be questioned according to the current international guidelines in 60 patients whose serum PSA level was < 2 ng/mL whereas they did not undergo prostatectomy*”. In our survey, ^68^Ga-PSMA-11 PET/CT was positive in 45/60 = 75% of those cases, revealing an oligometastatic spread (1–3 foci) in 23% of cases. The approach of ANSM, which granted or refused nATUs after analysing the whole medical records of those patients, was not based on a mechanical application of a cut-off value but on the agreement between several specialists: the referring oncologist or urologist, the nuclear medicine physician of the PET centre and the independent clinical expert of ANSM. In view of the favourable positivity rates in those two clinical settings, a revision of those international criteria for BCR is advised, in relation with the advent of ^68^Ga-PSMA-11 PET/CT. The objective of those criteria for BCR is to select patients in order to localise recurrence at the earliest possible.

Concerning ^18^F-fluciclovine, which was granted a MA in EU after our nATU procedure has been launched, no study showing a superiority of ^18^F-fluciclovine over FCH was available, only one comparative study vs. ^11^C-choline performed at the centre of Professor Fanti in 50 BCR patients [8]. The ANSM experts took this new tracer into account and advised that it may be accepted as an alternative to FCH for the prerequisite of nATU. In contrast, performing both FCH and ^18^F-fluciclovine will not be justified, to avoid further delay for a PSMA-11 nATU and to prevent an increased patient’s radiation exposure. During the whole survey, only one nATU was required on basis of a negative ^18^F-fluciclovine PET/CT and granted; in this patient, ^68^Ga-PSMA-11 was taken-up by a pelvic lymph node, treated by targeted radiotherapy and 6 months of hormonotherapy, which resulted in no recurrent rise in serum PSA level 1 year later. This case was included in our original manuscript submitted for publication but was removed from its revised version, as the reviewer advised to enhance the homogeneity of our series. None of the PET centres which performed ^68^Ga-PSMA-11 PET/CT during the survey period replaced FCH by ^18^F-fluciclovine in routine work-up of BCR. Since our survey reports on PET/CT with ^68^Ga-PSMA-11, and not on FCH PET/CT, the absence of a cohort of patients selected on basis of non-conclusive ^18^F-fluciclovine PET/CT reflects the practice of those French nuclear medicine specialists, which may be considered as a complementary information, but not *a weakness* of the survey.

About the acceptance by ANSM of the positivity rate (PR) as the end-point for efficacy, without evidence that the abnormal foci actually correspond to prostate cancer lesions, we already quoted in our article 10 studies of large cohorts, including recent ones, based on the same end-point. In case of occult recurrence, any positive focus on PET/CT should be checked and better characterised before the management decision is taken. This is why PR is important. As mentioned in the article, on a per patient level, the difference between PR and the rate of true-positive results, corresponds to the rate of false-positive results since no true-negative result is expected. The recent study of Fendler et al. [9] responds to the relevant objection of F Giammarile and S Fanti against PR: *“what if the new tracer produces a very high number of false positive findings?”* In the Fendler’s study the evaluation of diagnostic performance was based on a composite standard of truth; the patient-based PR was 475/635 = 75% and the patient-based rate of false-positive results with ^68^Ga-PSMA-11 was 17/217 = 8%, only one tenth of the PR. Therefore, assessing the PR in a large series as ours seams a reasonable option to reflect the actual diagnostic performance of this tracer. Concerning the wording, we agree that “positivity rate” better describes this parameter than “detection rate” which is ambiguous: detection of subsequently characterised cancer lesions or of any anomaly? In the original manuscript, we used the term “detection rate” that we replaced in the text by “positivity rate” according to the suggestion of the reviewer. We maintained “detection rate” in the title, as it has been and is still being used far more frequently in the published studies, which makes this expression currently more familiar to the readers.

Regarding the close collaboration of our Medicine Agency with the scientific societies, it is in the interest of both parties and of patients for a rapid availability of innovative and relevant diagnostic agents. One of the most important ANSM’s objectives is openness to stakeholders, including scientific societies. A close collaboration exists with the French concerned societies, both SFMN (the French Nuclear Medicine society) and AFU (the French Association of Urology), which is expected to be enhanced in next years.

In conclusion, this survey was an opportunity to report, in a large series of patients, on the added value of ^68^Ga-PSMA-11 in BCR at any serum level of PSA, but also on its PR according to the PET acquisition protocol, the potential metastatic sites and various determinants, that may be helpful in designing future studies.
